# Metabolite of chiral cycloxaprid in solvent and in the raw of Puer tea

**DOI:** 10.1016/j.fochx.2023.100654

**Published:** 2023-03-29

**Authors:** Hen Tian, Jiao Zhang, Tao Lin, Qiwan Li, Xiangzhong Huang, Hongcheng Liu

**Affiliations:** a Institute of Quality Standard and Testing Technology, Yunnan Academy of Agricultural Science, Supervision and Testing Center for Farm Product Quality, Ministry of Agriculture, Kunming 650223, PR China; b Key Laboratory of Chemistry in Ethnic Medicinal Resources, Yunnan Minzu University, Kunming 650500, PR China

**Keywords:** Metabolite, Cycloxaprid, Raw Puer tea processing

## Abstract

Cycloxaprid (CYC) with a chiral oxabridged cis- structure contains a pair of enantiomers. Enantioselective degradation, transformation and metabolite of CYC was performed in different solvents under light and raw Puer tea processing. The results showed that cycloxaprid enantiomers in acetonitrile and acetone was stable over 17 day, however the transformation of *1S, 2R*-(–)-cycloxaprid or *1R, 2S*-(–)-cycloxaprid was founded in methanol. The fastest degradation of cycloxaprid occurred in acetone under light, the metabolites were founded with retention times (T_R_) at 34.83, 15.78 min, which mainly was via the reduce reaction of NO_2_ to NO, and rearrange reaction to tetrahydropyran. Degradation pathways were via the cleavage of the oxabridge seven member ring and the whole C ring. However, the degradation pathway under raw Puer tea processing was via the cleavage of whole C ring and the cleavage of oxabridge seven member ring and reducing NO_2_, then it underwent an elimination of nitromethylene and rearrange reaction. This pathway of Puer tea processing was firstly founded.

## Highlights

• The present study was performed with enantioselective degradation, transformation and metabolite of CYC in different solvents under light and raw Puer tea processing.
• This degradation pathway in acetone under and Puer tea processing was firstly reported.

## Introduction

Neonicotinoids is a valuable synthetic insecticides piercing-sucking pests of for tea protection (Tomizawa & Casida, 2003). They are used increasingly and occupied 24% of insecticides in world market (Jeschke et al., 2011). In 2013, the European Commission prohibited the use of three neonicotinoide insecticides (Thiamethoxam, imidacloprid and thiamethoxam) that might affect the life cycle of bees, which is urgent to assess other neonicotinoid’s food safety risk (Chen et al., 2017).

Li et al. 2011 reported that a new neonicotinoid insecticide of cycloxaprid (CYC) has been synthesized and applied in China. It is different from traditional neonicotinoids, which act as agonists of native and recombinant nicotinic acetylcholine receptors (nAChRs) (Liu and Casida, 1993, Matsuda et al., 1998, Nishimura et al., 1994, Tomizawa and Casida, 2003). Cycloxaprid is chiral compounds, which contains a chiral oxabridged *cis*-configuration (C ring) leading to a pair of enantiomers, *1R,2S*-cycloxaprid and 1S,2R-cycloxaprid.

In recent years, chiral pesticides is the main focus of great attention at stereoselective biological activities and environmental processes of isomer selectivity. Chiral pesticides are commonly used as racemic mixtures and their stereoisomers are often degraded stereoselectively in food. Previous studies shown that the stereomiosmer had been found in soil or food. Liu et al. 2022 found the enantioselectivity of cycloxaprid in raw or ripen Puer tea processing. Zhang et al. 2013 observed stereoselective uptake and translocation of cycloxaprid in edible vegetables from roots. However, Chen et al. 2017 demonstrated that there was non stereoselective preference for cycloxaprid enantiomer in aerobic soils, so the enantioselectivity was effected by different matrix and the result was different. The degradation of pesticide is found the metabolite which maybe result in high toxicity in environment or food. Liu et al. 2015 identified and tracked 11 and one unknown transformed product in flooded and anoxic soil. Chen et al. 2017 reported three mainly metabolites included cleavage of the oxabridged seven-member ring and C—N between chloropyridinyl methyl and imidazalidine ring, carboxylation of the alkene group, and hydroxylation of imidazolidine ring in aerobic soil. Hou et al. 2017 studied photodegradation of CYC and analyzed 25 photodegradation products were identified via UPLC-TOF-MS/MS in water.

**Fig. 1:**
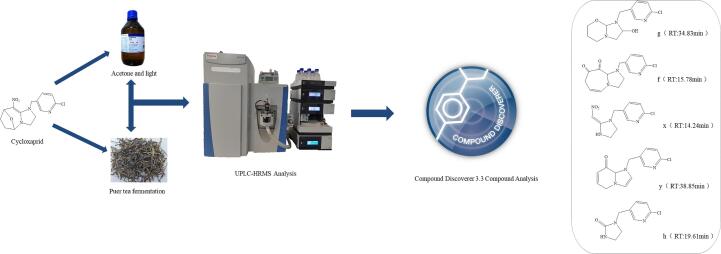
The degradation pathway and metabolite of cycloxaprid in Puer tea processing.

Puer tea is the most famous tea in China. Because its special processing, the Puer tea is fermented by traditional technology in long-distance transport with consign for horse. Previous our studied that one mainly metabolite was reported in Puer tea processing (Liu et al., 2022) but, the relevant information of degradation pathway and metabolite of CYC was still incomplete (Fig. 1). Therefore, it is important to obtain the degradation productions and pathway of CYC. The present study was to use racemate CYC to characterize the degradation in raw Puer tea processing, analyzed its metabolites and proposed degradation pathways, which is also significant for the fate of other new pesticides in the food.

## Materials and methods

### Reagent

A racemic cycloxaprid was provided by Beilinwei technology Ltd. (Beijing, China). Cycloxaprid powder 25 % was supplied by shanghai shengnong pesticide Co Ltd (Shanghai, China). The purified materials of C18, PSA, Carb were supplied by Dima technology co. Ld. (Beijing, China).

The stock solutions were produced by dissolving the racemic cycloxaprid in acetonitrile. All solutions were stored in a refrigerator at − 18 °C. HPLC-grade acetonitrile and methanol were provided by Tedia Company Inc. (OH, USA). The initial dose of cycloxaprid 0.15 g/L was used with 0.6 g of cycloxaprid power completely dissolved into 1 L water.

High seed centrifuge of TGL-10B was supplied by Anting co. Ld. (shanghai, China); The voter mixer of QL-866 was from Linbei intrument co. Ld. (Jiangsu, China); The high speed of DFT-100A 100was from Linda machine co. Ld (Zhejiang China).

## Methods

Incubation experiments were performed to investigate the enantioselective racemic cycloxaprid and the metabolism in raw Puer tea processing. Five grams of raw Puer tea are sprayed with 0.15 g/L the formulation product solution. A nontreated control was also included. The enantioselective degradation is express as enantiomer fraction (EF) and determined by LC-MS/MS. The metabolomics are analyzed by LC-HRMS.

To research stability and transform, *1R,2S* **-**cycloxaprid or *1S,2R*-cycloxaprid (1 mg/L otpical pure) are respectively tested with different solutions (methanol, acentrinole, acentone), with light or dark.

To research dissipation during light or dark, three hundred grams of raw Puer tea are sprayed with 0.15 g/L the formulation product solution. One sample is placed with black plastic bag on dark room, the other sample is not protected under air temperature. The samples are collected at intervals time on 0 (2 hr), 1, 3, 5, and 14 day. The residues amount expressed as dry sample.

### Obtaining enantiopure standards: *1R,2S* -cycloxaprid and *1S,2R*-cycloxaprid

The enantiopure of *1R,2S* -cycloxaprid and *1S,2R*-cycloxaprid is separated by semi-preparative HPLC. The 4.8 mg/mL of cycloxaprid in acetonitrile solution was separated by using a Chiralpak AG (amylose tris (3-chloro-5-methylphenylcarbamate) as stationary phase, 250 × 4.6 mm i.d., 5 μm, Daicel Ltd. JP). Mobile phases are H_2_O and acetonitrile (55: 45), respectively. The flow rate was 1.0 mL/min. the injection volume is 40 μL.

### Samples preparation

Two gram of tea sample were exactly weighed, then 10 mL water, 10 mL acetonitrile were added. After the mixture was vortexed, and added 4 g NaCl. The tube with vortex mixer was shaken vigorously at 1 min. The mixture was centrifuged at 5000 rpm for 5 min. The upper layer solution was separated and added by 100 mg PSA, 100 mg C18, 50 mg carb and 300 mg anhydrous MgSO_4_. After shaking and centrifugation at 5000 rpm for 3 min, 0.5 mL of the upper layer was separated and filtered through 0.22 μm filter for UPLC-HFMS or UPLC-MSMS.

### UPLC-HRMS analysis

Sample analysis was achieved in an Ultra Performance Liquid Chromatography – Q Exactive high resolution Mass Spectrometry (Thermo Fisher Scientific, Rockford, IL, U.S.A.) system. The chromatogram seperation was obtained by Hypersil GOLD (100 × 2.1 mm, 1.9 μm), and the temperature is maintained at 30 °C. The mobile phase is comprised of 80% 0.1% formic acid, 1 mmol ammonium with water/20% acetonitrile at a constant flow of 0.3 mL/min. The gradient mobile phase program (min/%B) was 0–3/3–5, 3–10/35–55, 5–10/55, 10–20/10–15, 20–30/15–18, 30–50/20–40, 50–53/40, 53–60/40–95, 60–61/95–3. The injection volume is 1 μL.

The instrument was tuned in the positive ESI mode (3.8 kV of spray voltage, 325 °C of capillary temperature, 350 °C of probe heater temperature and 60 V of SLens). The instrument was calibrated using positive calibration solutions. The FS/DIA mode was used. In FS, the scan range was *m*/*z* 50 –650; mass resolution at 140,000 FWHM; AGC target and maximum IT were set at 1.0 e^6^ and 100 ms, respectively. For DIA, the relevant parameters were set as follows: mass resolution: 140,000 FWHM; AGC target: 2 e^5^; maximum IT: 30 ms; Loop count: 12; MSX count: 1; Isolation window: 50 Da; stepped normalized collision energy (NCE): 20%, 40% and 60%. The spray voltage in positive and negative modes was set as 3.5 kV and 3.0 kV, respectively. The flow rate of sheath gas and aux gas was 45 and 10 (in arbitrary units), respectively. The software was used with TraceFinder 4.1 EFS and Compound Discoverer 3.3.

### UPLC-MSMS analysis

UPLC-MS/MS was used with tandem mass spectrometer AB 4500 (AB Sciex) consisted of a 1290 ultra-high-performance liquid chromatograph (Agilent technology). A Chiral amylose tris (3- chloro- 5-methylphenylcarbamate), 250 × 4.6 mm i.d., 5 μm was used and the column temperature was set at 30 °C. The mobile phase is comprised of 80% 0.1% formic acid, 1 mmol ammonium acetate with water/20% acetonitrile at a constant flow of 0.3 mL/min with 1 μL injection. The gradient mobile phase program (min/%B) was 0–10/35, 10–11/35–55, 11–20/55, and 21–25/95. Anion electrospray ionization mode was used, and multiple reactions monitoring (MRM) was utilized: Precursor ion was set at 323.1 (*m*/*z*), Product ion was set at 126.0*/138.1, 90.1 (*m*/*z*), Delustering/Focusing potential was set at 77 (V), and Collision energy was set at 40/35, 76 (V). The temperature and flow rate of drying gas (N2) was set at 550 °C and 8.0 L/min, respectively. The nebulizer pressure was set at 20 Pa.

### Quality control

Cycloxaprid at three concentration levels (1, 10, 100 µg/kg) with matrix solution is used for limit of detection limit and recovery test. The chromatogram of spiked sample and elution order of racemic cycloxaprid was determined by online optical rotation. The more separation resolution was obtained by the method than Zhang et al. 2013 reported. No cycloxaprid is residue in the blank sample. The mean recoveries are in the range of 76–108 % for racemate of cycloxaprid, respectively. The corresponding relative standard deviation was 4.6–9.2%. The linearity is the range of 1–100 µg/L with coefficient, R^2^ > 0.999. The limit of detections (LODs) with three times signal-to-noise (S/N) ratio, and the limit of quantify (LOQ) with ten times S/N ratio is 0.5 μg/L, 0.5 μg/kg respectively.

### Data analysis

The data was obtained by triplicate samples. The results showed as the means with standard errors (means ± STD) using statistical analysis software. The significant difference (p = 0.05) among treatments was determined by one-way analysis of variance.

## Results and discussion

### The stability of optical pure compounds in different solvent

**Fig. 2:**
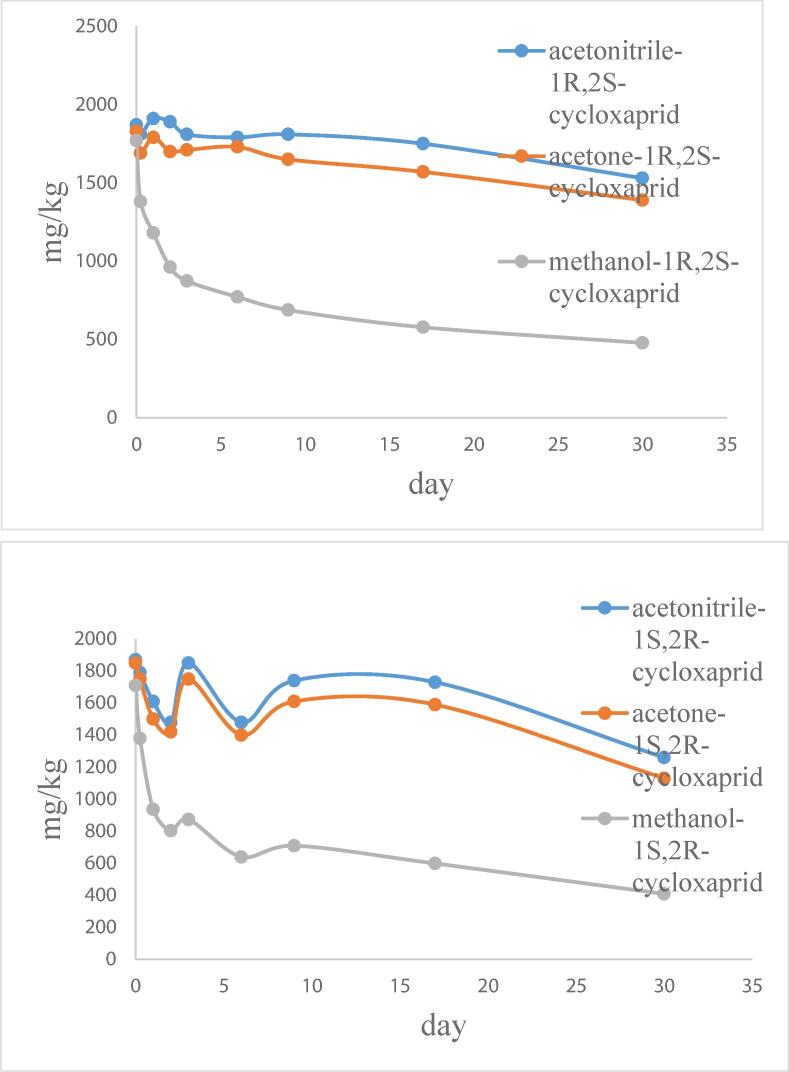
The stability of optical pure compounds (1S, 2R-cycloxaprid or 1R, 2S-cycloxaprid) in three solvents.

The stability of optical *1R, 2S*-cycloxaprid and *1S, 2R*-(–)-cycloxaprid was tested in three solvents. The results showed that *1R, 2S*-cycloxaprid or *1S, 2R*-(–)-cycloxaprid in acetonitrile and acetone was stable over 17 day. However, optical pure of *1R, 2S*-cycloxaprid was not stable in methanol, which was quickly decreased in methanol. But the racemate CYC was stable, the result showed that the transformation of *1S, 2R*-(–)-cycloxaprid was formed in Fig. 2. A similar result is observed with other optical pure compounds (Zhang et al., 2013).

The parent compound of racemate CYC in solvent was more unstable under light than under dark. Hou et al. 2017 reported the pathways of the photoreaction process of CYC was hydroxyl radicals reaction. The degradation of CYC in three solvents was distinctly decreased under light. The CYC in acetone was not detected over 9 day, but an amount of residues in methanol, acetonitrile were founded. This suggest that the degradation of cycloxaprid was catalyzed by light radicals and speed by acetone solvent.

### Formation of metabolites in acetone under light

The metabolite research is major limited with the relatively small number of metabolites commercially available as pure standards and structure determination by NMR or X-ray crystallography, therefore the large number of metabolites with unknown chemical structures that remain to be identified and characterized by High resolution mass spectrum (Ivana et al. 2019). Novel computational tools can predict MS fragmentation patterns in databases (Cajka et al., 2017, Kind et al., 2018), which has been shown that fragmentation spectra can be simulated with quantum chemical and molecular dynamics methods (Wishart et al., 2018).

**Fig. 3:**
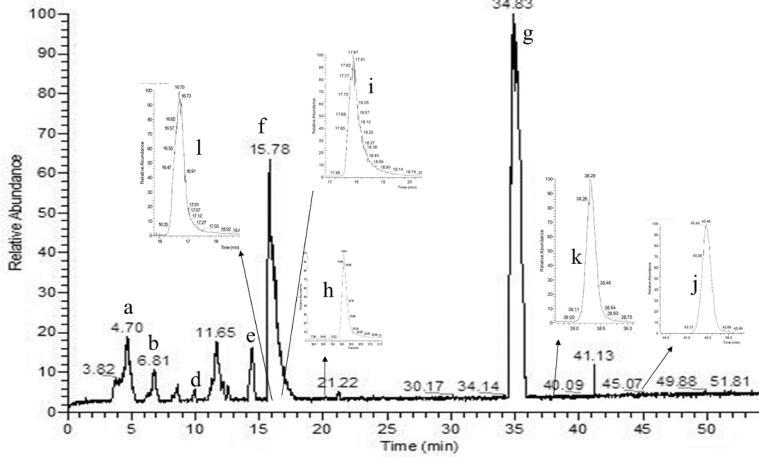
The total ion chromatogram of cycloxaprid in acetone under light.

Fig. 3 shows the chromatogram of main metabolites under light with acetone at 13 day after cycloxaprid spiking. There were two large peaks with the retention times (T_R_) at 34.83(g), 15.78(f) min, and four main peaks were named as a, b, d, e. We were achieved metabolite information using a combination of different tools. The metabolite compounds were identified based on accurate mass and retention time using the NIST17, HMDB 20, GNPS 21, GMD and the Lipid MAPS libraries, which obtained the molecular structure. Then for MSI annotations, we used mzCloud (online second mass spectrometry),ChemSpider (online first mass spectrometry) accurate mass search services for validating and putatively annotating the metabolite feature using mass spectrometry (Table 1).

**Table 1:** Mass spectrometry data for the identification of cycloxaprid and its metabolites in acetone under light.

Product	tR (min)	Formula	Mw	Chemical structure	Mw (calculated)	ESC(+) MS, *m*/*z*	ESI(+)MS2, *m*/*z* (relative abundance %, loss)
Parent	17.72	C_14_H_15_ClN_4_O_3_	322.0833		323.09054	323.09033	323.09033 (60, M + H); 325.08752 (20,M + H + 2); 289.08527 (14,M-O2H2); 277.09681 (36, M-NO_2_); 276.08992 (36);248.09483 (27, M- NO2 –CO); 234.07930 (22); 220.06371 (12);182.09239 (20);151.08655 (40, M- NO2 + ClC5H3NCH); 138.06631 (16);126.01065 (100, M-OC3H6-N2C3H2O); 128.00773 (30);123.09184 (22, M- CO - ClC5H3NCH);
g	34.83	C_12_ H_16_ O_2_ N_3_ Cl	269.09256		270.10038	270.10032	270.10029 (2, M + H); 272.09685 (1, M + H + 2); 212.05838 (6, M-OC3H6); 197.04748 (10, M-OC3H6-NH); 183.03189 (16, M-OC3H6-NH-CH2); 126.01067 (100, M-OC3H6-N2C3H2O); 128.00766 (24)
l	16.66	C_14_ H_15_ Cl N_4_ O_2_	306.08780		307.09563	307.09518	307.09518 (100,M + H); 309.09239 (22,M + H + 2); 289.08478 (4, M-OH); 276.08971 (4); 261.09000 (6);248.09475 (3); 220.06317 (6, M- OH-NO -C3H4); 208.06335 (4); 169.05252 (4); 164.08178 (4);149.07100 (2);136.08681 (6, M-OC3H6-NC3H2O); 126.01054 (52, M-OC3H6- N2C3H2O); 121.07638 (4);
j	45.66	C_14_H_16_ClN_3_O	277.09764		278.10547	278.10544	278.10544 (14,M + H); 280.10185 (6,M + H + 2); 236.09562 (12); 211.06319 (42); 149.02374 (100, M-C6H7NCl); 136.07554 (24);126.01062 (80);
k	38.27	C_14_ H_13_ Cl N_4_ O_2_	304.07215		305.07998	305.07922	305.07922 (36, M + H); 307.07768 (12, M + H + 2); 288.07707 (42, M-OH); 261.08387 (22);259.08668 (100, M-O-NO);258.07942 (50,M-NO2);243.05620 (4);232.06386 (12);224.11781 (14);216.04722 (4);167.03752 (4);147.09109 (4);133.07571 (4);126.01064 (48);
b	6.81	C_14_H_16_O_3_N_3_Cl	309.08747		310.09530	310.09482	310.09482 (38, M + H); 312.09258 (10, M + H + 2); 196.06344(100, M-C5H6O3); 198.06034(24); 160.08690(8); 126.01055(55, M-C8H9O2N3);
f	15.78	C_14_H_14_ClN_3_O_2_	291.07691		324.11095	324.11063	324.11063(29, M + H + MeOH); 306.10770(8, M-H2O + MeOH);196.06357 (100, M-C5H4O2); 126.01058 (48, M-C8H9O2N3); 101.06007 (20);
i	17.51	C_14_ H_14_ Cl N_3_ O	275.08199		276.08982	276.08973	276.08974 (100, M + H); 278.08365 (16, M + 2); 248.09497 (28, M-CO); 234.07896 (10,M-O-C2H2); 220.06340 (12,);208.06361 (16);193.03998 (4,M-C5H7O);151.08654 (40);126.01065 (87, M-C8H10N2O);123.09184 (18);
r	12.09	C_14_ H_16_ Cl N_3_ O_2_	293.09256		294.10038	294.10000	294.10000(100,M + H);296.09686(23,M + H + 2);264.05134(1,M-CH2O);238.07403(10,M-CH2O-C2H2);235.02437(4);196.06344(1, M-C5H6O2);169.05281(3); 149.02268(1); 126.01063(38, M-C8H10N2O); 128.00760(10);
a	4.70	C_9_H_10_N_3_Cl	195.05578		196.06360	196.06368	196.06371(72, M + H); 198.06058 (15, M + H + 2); 160.08694 (8, M-Cl); 126.01075(100, M-N2C3H6); 128.00772(22);90.03409 (2)
h	19.61	C_9_H_10_ClN_3_O	211.05069		212.05852	212.05819	212.05821 (99,M + H); 214.05520 (20,M + 2); 176.08186 (2,M-Cl); 130.02320 (21);128.02615 (100,M-C3H4N2O); 126.01055 (20);99.05565 (32);
O	4.02	C_10_ H_12_ Cl N_3_ O	225.06634		226.07417	226.07370	226.07426(96,M + H); 228.07117(24,M + 2); 196.06368(28, M-CH2O); 198.06073(8,M-CH2O + 2); 160.08684(8,M- M-CH3O-Cl); 130.02317(4); 128.00775(22); 126.01075(100,M-C4H8N2O);
d	10.02	C_10_*H*_10_ Cl N_3_ O_2_	239.04561		240.05343	240.05316	240.05316(52,M + H);242.05013(12,M + H + 2);126.01063(100, M-C4H6O2N2); 128.00759 (22)
P	12.25	C_10_ H_12_ Cl N_3_ O_2_	241.06126		242.06908	242.06847	242.06849(38,M + H); 244.06528(10,M + 2); 224.05838(6,M-H2O); 214.07361(14); 199.04403(8); 197.04720(28, M-COOH); 169.05256(2);143.03676(2); 128.00752(22); 126.01056(100, M-C4H8O2N2);
e	14.36	C_11_H_14_ Cl N_3_ O_2_	255.07691		256.08473	256.08439	256.08439(22,M + H); 258.08192(6,M + H + 2); 238.07397(10, M-H2O); 228.08956 (12, M-CO); 211.06319(8, M-H2O-CHN); 196.06353(42, M-C2H4O2); 126.01063(100, M-C5H10N2O2);

The fragment pathways of CYC were three in Fig. 4, One possible pathway was via the cleavage of the oxabridged ring (C ring). The second possible route was oxidated the C ring. The third possible route was removed of chloropyridinyl (A ring). The pathway was similar with Hou et al. 2017 reported the pathway of photodegradation in water.

**Fig. 4:**
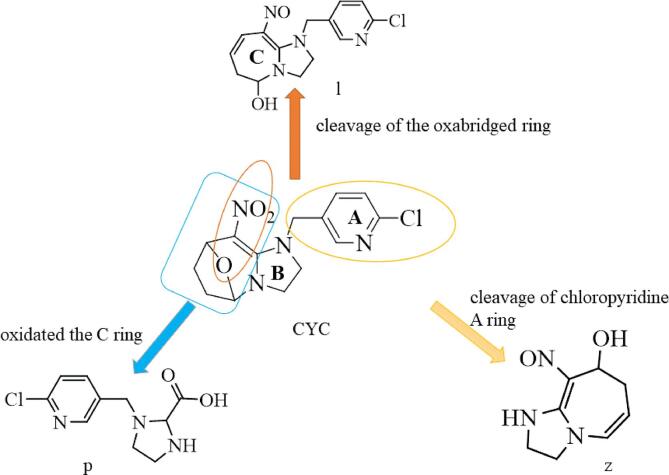
The fragment pathway of cycloxaprid.

**Fig. 5:**
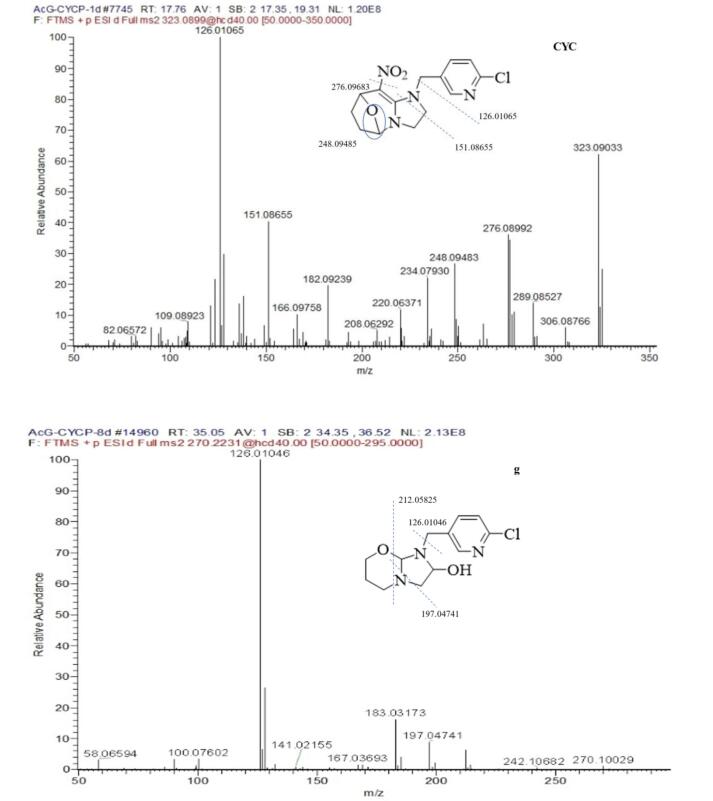

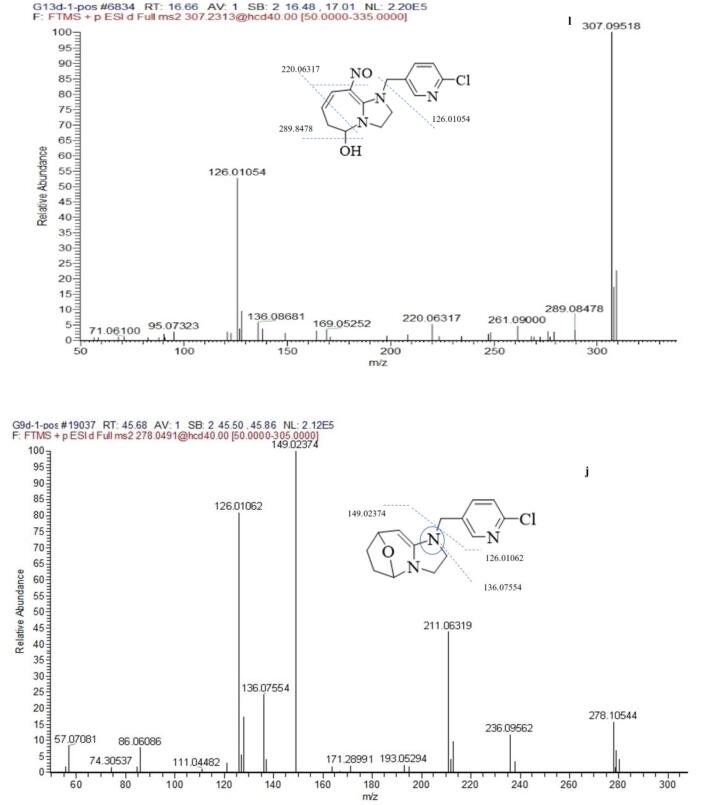

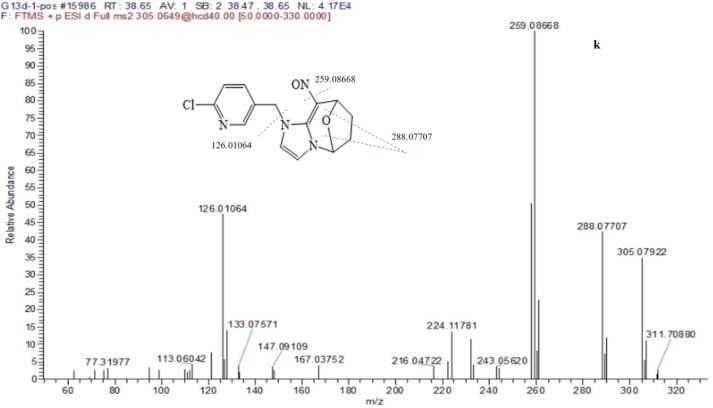
The ms/ms spectra of cyc and main metabolite under light.

The peak at the retention time of 17.72 min with the molecule ion at *m*/*z* 323.09033 [M + H]^+^ and chlorine isotopic ion at *m*/*z* 325.08752 [M + H + 2]^+^ was CYC. The base peak of characteristic ion at *m*/*z* 126.01065 and chlorine isotopic ion at *m*/*z* 128.00773 corresponding to chloropyridinylmethyl, which could be found in other metabolite. The other major fragments at *m*/*z* 276.09681, 248.09485, 151.08655, 123.09183 exhibited the loss of NO_2_, NO_2_ + CO, NO_2_ + ClC_5_H_3_NCH, and CO + ClC_5_H_3_NCH, respectively. The Fig. 5 showed MS/MS spectra of CYC and main metabolite.

Metabolite g was the major metabolite in total ion chromatogram. The product ions 270.10032 [M + H]^+^ containing odd nitrogen and chlorine isotopic ion ^37^Cl, corresponding to the formula of C_12_ H_16_ O_2_ N_3_ Cl. The product ions 212.05803, 197.04741 produced via the loss of OC_3_H_6_ and OC_3_H_6_-NH, which was identified as 1-[(6-chloropyrid-3-yl) methyl]-2-hydroxyl-imidazoline-tetrahydropyran, which probably is a rearrange reaction from metabolism j.

Metabolite k, l, j was probably a leading compounds of g. The l of product ion 307.09518 and the k of 305.07922 was reduced oxygen from the NO_2_ of CYC, and the three metabolites containing product ions *m*/*z* 126.01054 resulted from the cleavage of the C—N bond linking the A + B rings. The retention time of metabolite j and k was at 45.66, 38.27 min respectively, but metabolite l was at 16.66 min, which indicated that metabolite l was polar compound with alcohols and metabolitea j, k were low polar compound with oxo-bridge structure in Fig. 4.

The mass spectrum of a that appeared at 4.70 min in the chromatogram yielded mass peaks of *m*/*z* 196.06368 [M + H]^+^; the base peak with *m*/*z* 126.01074 [M-N_2_C_3_H_6_]^+^; Metabolite f that appeared at 15.78 min in the total ion chromatogram exhibited an [M + H + MeOH]^+^ ion at *m*/*z* 324.11063, which corresponds to the formula of C_14_H_14_ClN_3_O_2_, the ion *m*/*z* 306.10770 with cleavage of H_2_O. The base peak of *m*/*z* 196.06357 (a) and the ion of *m*/*z* 126.01058 (a) which suggested the A and B rings were completed. Characteristic ion 196.06357 and 126.01058 also appeared in the fragments of metabolite b, f, and r. Metabolites of b, f, r also had been detected by photodegradation products of CYC (Hou et al. 2017).

Metabolite e, with *m*/*z* 238.07397 (10, M-H_2_O) and *m*/*z* 228.08956 (12, M-CO), which suggested that the structure contained simultaneously hydroxyl and aldehyde group.

Metabolite p, with an *m*/*z* 242.06847, which corresponds to the formula of C_10_H_1_°ClN_3_O_2._ Characteristic ion 126.01056 appeared in the fragment of metabolite d, o, a. Those compounds had been reported by Hou et al. 2017.

## Proposed degradation pathway under light

**Fig. 6:**
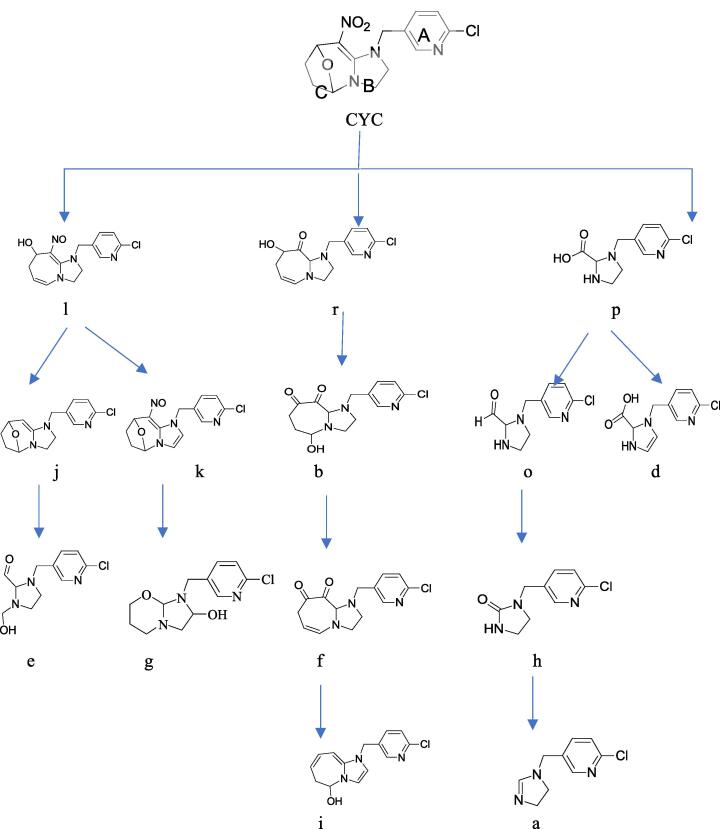
The hypothetical degradation pathway of cycloxaprid in acetone under light.

Based on the identified degradation products, the possible photodegradation pathways in aqueous solution under light irradiation were proposed. The tentative photodegradation pathways of CYC were shown in Fig. 6. The degradation of CYC in acetone solution under light via three possible pathways.

One main degradation pathway was via the reduce reaction of NO_2_ to NO, and rearrange reaction to tetrahydropyran, which was not previously reported (Fig. 6) Two degradation pathways were via the cleavage of the oxabridge seven member ring and the whole C ring, resulting in the formation of primary compound. These pathway was very similar to that reported in the photodegradation of CYC under environment. (Hou et al. 2017).

### Formation of metabolites in raw Puer tea

**Fig. 7:**
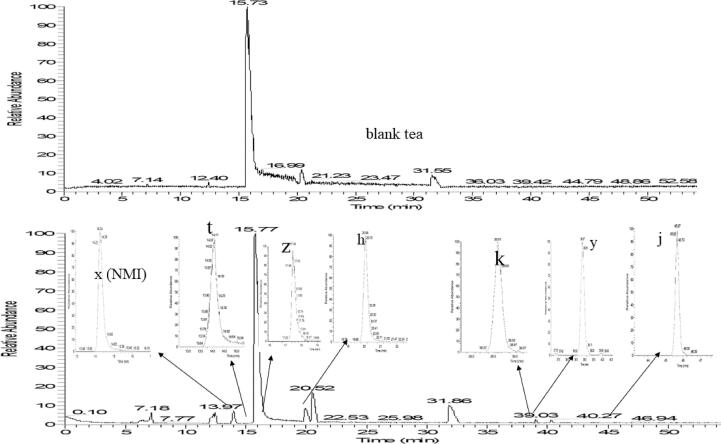
The total ion chromatogram of raw Puer tea and cycloxaprid spiked.

To obtain the transformation, the conversion of cycloxaprid is quickly generated by sun light and the normal fermentation in air temperature. Fig. 7 shows the main metabolites under raw Puer tea processing at 13 day after cycloxaprid spiking and blank. There were three obvious peaks with the retention times (T_R_) at 13.97 (x), 19.61(h), 39.09 (y) min, and the diversity peaks were similar with these peak on metabolites in acetone under light (metabolite of l, k, j, i).

Metabolite × was the major metabolite reported by our previous results (Liu et al., 2022). The metabolite and standard were simultaneously analyzed by LC-HFMS, and their retention time, molecular ion peak, and fragmentation pattern were in agreement with the synthesized structure standard of 2 -chloro-5 -[[-2 -(nitromethylidenc) imidazodin-1 -yl] methyl] pyridine (CAS 10336–63-4). A similar result was also observed by Chen et al. 2017. The result indicated that most CYC might transform to the active intermediate x. Furthermore, Shao et al. 2010 showed that CYC may be a slow-release reservoir for (nitromethylene) imidazole (x), which was considered as the final active ingredient.

**Fig. 8:**
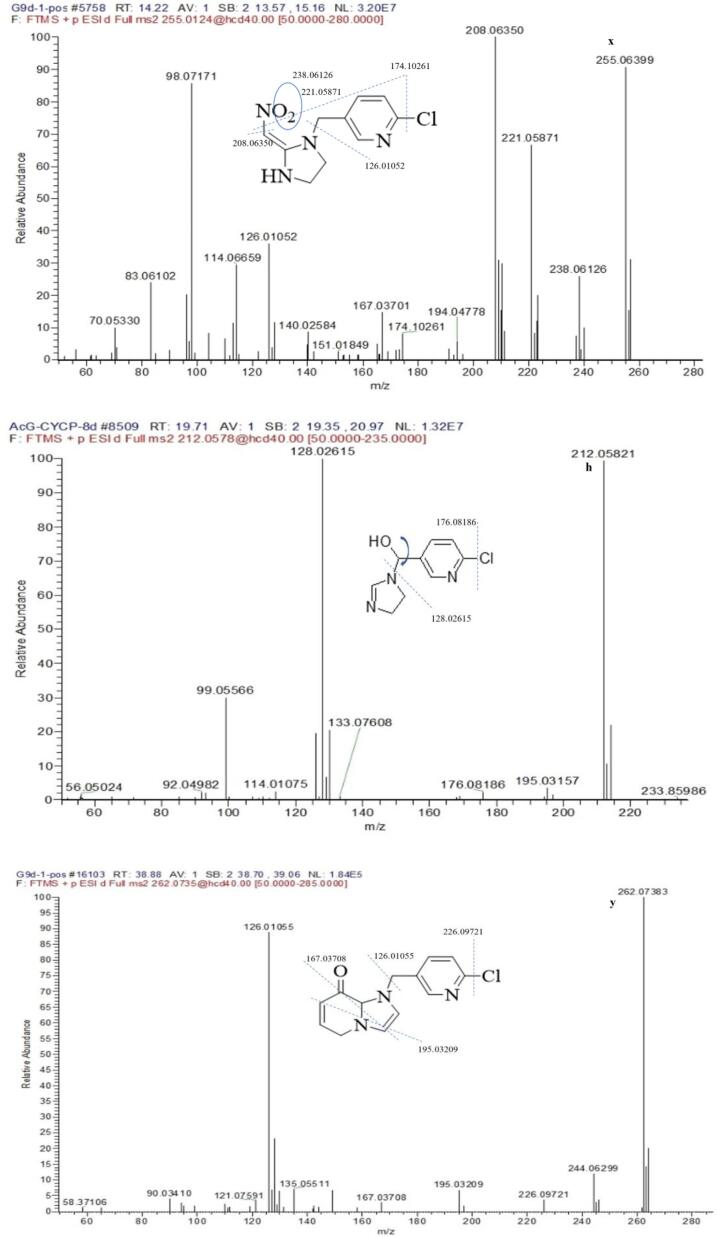

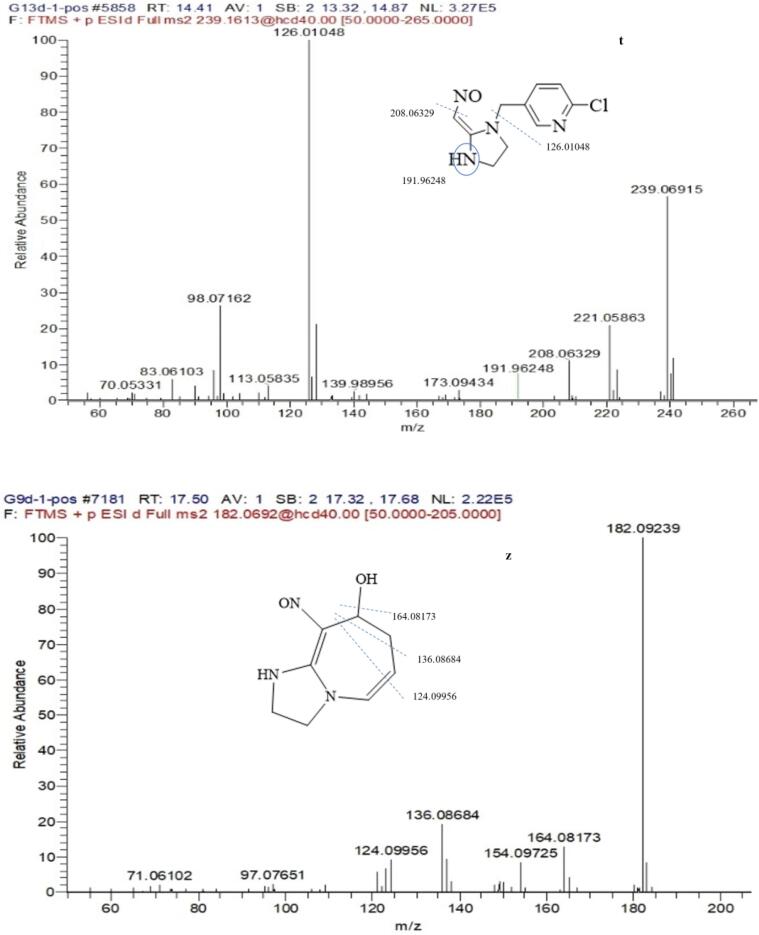
The ms/ms spectra of main metabolite in puer tea processing.

The mass spectrum of h that appeared at 19.61 min in the chromatogram yielded mass peaks of *m*/*z* 212.05819 [M + H]^+^, which corresponded to the formula of C_9_H_10_ON_3_Cl. The Fig. 8 showed MS/MS spectra of main metabolite. The base peak was more different with *m*/*z* = 128.02614 than with characteristic ion *m*/*z* = 126.01055. The result suggested that the pyridine ring was added hydroxyl group and oxygen rearrange reaction.

Metabolite y that appeared at 38.85 min in the total ion chromatogram exhibited an [M + H]^+^ at *m*/*z* 262.07383, which corresponds to the formula of C_13_H_12_ClN_3_O, *m*/*z* 244.06299 [M-H_2_O]^+^,which showed that the compound contain hydroxyl or carbonyl group. The chromatogram condition exhibited the low polarity compound with long retention time (38.85 min), so the chemical structure was deducted in Tab 2.

Metabolite t that appeared at 14.41 min in the total ion chromatogram exhibited an [M + H]^+^ at *m*/*z* 239.0691 and chlorine isotopic ion *m*/*z* 241.06527, which corresponds to the formula of C_10_H_11_ClN_4_O. The fragment *m*/*z* 208.06329 [M-NO]^+^ showed that the B ring contain NO group, the even nitrogen rule indicated the complete A ring at *m*/*z* 191.96248 [M-NO-NH2]^+^.

Metabolite z was overlapped by tea matrix at 17.51 min exhibited an [M + H]^+^ at *m*/*z* 182.09239, which corresponds to the formula of C_8_H_11_N_3_O_2_. The fragment *m*/*z* 164.0817 remove H2O, and the fragment *m*/*z* 136.08684, 124.09956 removed C_2_H_4_, C_3_H_4_ which showed still the whole frame of B ring and C ring.

### Proposed degradation pathway under Puer tea processing

One degradation pathway was via the cleavage of whole C ring, resulting in the formation of mainly degradation compound X, which had been reported as the final active ingredient (Chen et al., 2017, Hou et al., 2017,; Liu et al. 2022). Metabolism X also underwent an elimination of nitromethylene and methylation of the imidazolidine ring, which produced h and w.

**Fig. 9:**
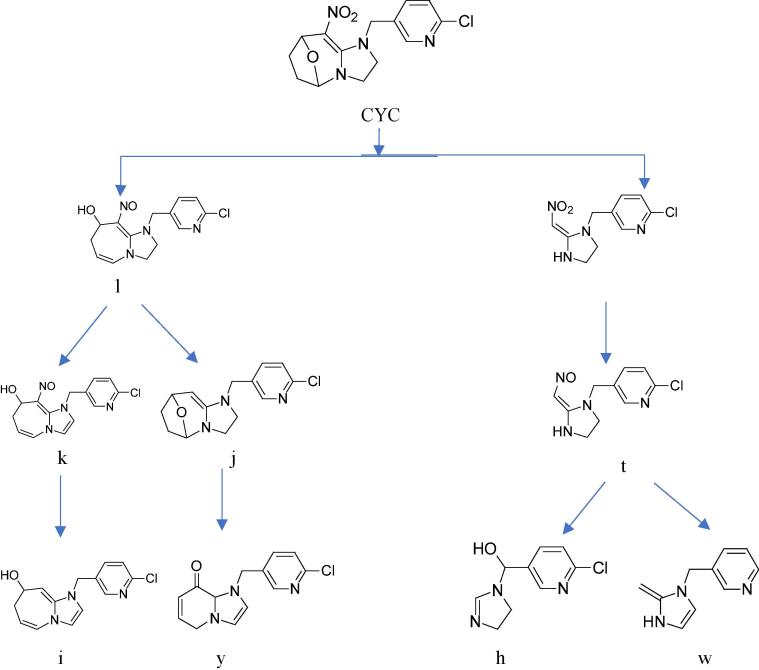
The hypothetical degradation pathway of cycloxaprid in raw Puer tea processing.

The other degradation pathway was via the cleavage of oxabridge seven member ring and reducing NO_2_, resulting in the formation of metabolism l, which was similar to degradation in acetone under light. Metabolism l also underwent an elimination of nitromethylene and rearrange reaction, which produced y. This pathway in Fig. 9 was very different to photodegradation in water (Hou et al., 2017) or our previously reported results (Liu et al., 2022). Compared to the photolysis, the metabolite of CYC was more complex in Puer tea processing. This study can provide insights to the fate of pesticide in the Puer tea processing.

## Conclusion

The enantioselective degradation, transformation and metabolite of CYC in different solvents under light and raw Puer tea processing was detected. Chiral cycloxaprid in acetonitrile and acetone was stable, however the transformation of *1S, 2R*-(–)-cycloxaprid or *1R, 2S*-(–)-cycloxaprid was founded in methanol. The fastest degradation of cycloxaprid occurred in acetone under light, the degradation pathway was via the reduce reaction of NO_2_ to NO, and rearrange reaction to tetrahydropyran. The degradation pathway under raw Puer tea processing was via the cleavage of whole C ring, and via the cleavage of oxabridge seven member ring and reducing NO_2_, then it underwent an elimination of nitromethylene and rearrange reaction (Table 2).

**Table 2:** Mass spectrometry data for the identification of cycloxaprid and its metabolites in raw Puer tea processing.

Product	tR (min)	Formula	Mw	Chemical structure	Mw (calculated)	ESC(+) MS, *m*/*z*	ESI(+)MS2, *m*/*z* (relative abundance %, loss)
Parent	17.72	C_14_H_15_ClN_4_O_3_	322.08272		323.09054	323.09033	323.09033 (60, M + H); 325.08752 (20,M + H + 2); 289.08527 (14,M-O2H2); 277.09681 (36, M-NO_2_); 276.08992 (36);248.09483 (27, M- NO2 –CO); 234.07930 (22); 220.06371 (12);182.09239 (20);151.08655 (40, M- NO2 + ClC5H3NCH); 138.06631 (16); 126.01065 (100, M-OC3H6-N2C3H2O); 128.00773 (30); 123.09184 (22, M- CO - ClC5H3NCH);
x	14.04	C_10_H_11_ClN_4_O_2_	254.05650		255.06433	255.06411	255.06399 (91,M + H); 257.06104 (32,M + H + 2); 238.06126 (28,M-OH); 221.05871 (68,M-O2H2); 208.06350 (100,M-HNO2); 174.10261 (8,M-NO2-Cl); 167.03701(14);140.02584 (8); 126.01052 (36); 114.06659 (30); 98.07171(86); 96.05608 (20); 83.06102 (24); 70.05330 (10);
T	14.41	C_10_*H*_11_ Cl N_4_ O	238.06159		239.06942	239.06915	239.06915 (58,M + H); 241.06527 (14,M + 2); 221.05863 (22);208.06329 (12,M-HNO); 191.96248(10, M-NO-NH2);173.09434 (3); 126.01048 (1 0 0); 98.07162 (26); 83.06103 (6);
Y	38.85	C_13_H_12_ClN_3_O	261.06634		262.07417	262.07383	262.07383 (100,M + H); 264.07060 (20,M + H + 2); 244.06299 (12);226.09721 (4,M-Cl);195.03209 (8,M-C4H5N);167.03708 (3,M-C5H5ON); 135.05511 (7);126.01055 (90,M-C7H8ON2); 90.03410 (2)
K	38.27	C_14_ H_13_ Cl N_4_ O_2_	304.07215		305.07998	305.07922	305.07922 (36, M + H); 307.07768 (12, M + H + 2); 288.07707 (42, M-OH); 261.08387 (22);259.08668 (100, M-O-NO);258.07942 (50,M-NO2);243.05620 (4);232.06386 (12);224.11781 (14);216.04722 (4);167.03752 (4);147.09109 (4);133.07571 (4);126.01064 (48);
Q	17.51	C_14_ H_14_ Cl N_3_ O	275.08199		276.08982	276.08973	276.08973 (100,M + H); 277.09464(24); 278.08497 (14,M + 2); 262.07416 (2, M-H2O); 248.09478 (28, M-H2O-CH2); 234.07907 (18, M-H2O-C2H4); 220.06322 (12, M-H2O-C3H6); 208.06347 (16,M-H2O-C4H6); 193.03991 (4); 151.08648 (28); 126.01063 (16); 123.09178 (14);
J	45.66	C_14_H_16_ClN_3_O	277.09764		278.10547	278.10544	278.10544 (14,M + H); 280.10306 (6,M + 2); 236.09562 (12); 211.06319 (42); 149.02374 (1 0 0); 126.01062 (80);80.06086 (8);57.07081 (8);
L	16.66	C_14_ H_15_ Cl N_4_ O_2_	306.08780		307.09563	307.09518	307.09518 (100,M + H); 309.09239 (22,M + 2); 289.08478 (4, M-H2O); 276.08971(4, M-NOH); 261.09000 (6); 248.09475 (3); 220.06317 (6); 208.06335 (4);169.05252 (4); 164.08178 (4); 149.07100 (2); 136.08681 (6); 126.01054 (52); 121.07638 (4);
H	19.61	C_9_H_10_ClN_3_O	211.05069		212.05852	212.05851	212.05821 (99,M + H); 214.05520 (20,M + H + 2); 176.08186 (2,M-Cl); 130.02320 (21); 128.02615 (100,M-C3H4N2O); 126.01055 (20); 99.05565 (32);
W	14.29	C_10_*H*_11_ N_3_	173.09475		174.10257	174.10264	174.10264(100,M + H);172.08679(29); 158.08382(3); 145.07596 (8, M-C2H3N); 133.07619 (6, M-C2H3N-CH); 118.06555 (6);
z	17.51	C_8_*H*_11_ N_3_ O_2_	181.08458		182.09240	182.09239	182.09239(100, M + H); 164.08173(14, M-H2O); 154.09725(8);136.08684(20, M-H2O-C2H4); 124.09956 (10); 123.09186(7); 121.07616 (6)

## CRediT authorship contribution statement

**Hen Tian:** Methodology. **Jiao Zhang:** Software. **Tao Lin:** . **Qiwan Li:** Conceptualization. **Xiangzhong Huang:** Validation. **Hongcheng Liu:** Writing – review & editing.

## Declaration of Competing Interest

The authors declare the following financial interests/personal relationships which may be considered as potential competing interests: Liu hongcheng reports article publishing charges was provided by Yunnan Academy of Agricultural Science. Liu hongcheng reports a relationship with Yunnan Academy of Agricultural Sciences that includes: employment. Liu hongcheng has patent No pending to Yes. employee.

## Data Availability

Data will be made available on request.
